# Le syndrome de Cri du Chat : A propos d’une observation

**Published:** 2012-01-12

**Authors:** Karim Ouldim, Imane Samri, Laila Bouguenouch, Hasna Hamdaoui, Ihsan El Otmani, Mohamed Hbibi, Sana Chaouki, Moustapha Hida

**Affiliations:** 1 Unité de Génétique Médicale et d’oncogénétique, Laboratoire centrale d’analyses Médicales, CHU Hassan II, Fès Maroc; 2 Service de Pédiatrie CHU Hassan II Fès, Maroc

**Keywords:** Cri du Chat, caryotype, génétique, chromosome, Maroc

## Abstract

Le syndrome du Cri du Chat (Cri du Chat syndrome, CdCS) est une anomalie chromosomique résultant d’une délétion de taille variable de l’extrémité du bras court du chromosome 5 (5p), incluant une région critique située en p15.2. Il représente une des délétions chromosomiques les plus fréquentes, son incidence dans la population générale est de 1/20 000 à 1/50 000. Les caractéristiques cliniques comprennent un cri monochromatique aigu, une microcéphalie, une dysmorphie cranio-faciale caractéristique évoluant avec l’âge et un retard mental et psychomoteur important. La taille de la délétion est variable, Le traitement est fonction des différents symptômes. Un remaniement chromosomique parental est retrouvé dans 12% des cas et la majorité des délétions responsables de la maladie du cri-du-chat surviennent de novo. Nous présentons une observation d’un syndrome du Cri du Chat, confirmé par caryotype métaphasique (46,XY,del(5)(p13) de novo). A travers cette observation nous mettrons à jour, les actualités scientifiques de ce rare syndrome, ainsi que la place des explorations cytogénétiques dans le diagnostic précis et le conseil génétique des syndromes dysmorphiques.

## Introduction

Le syndrome du Cri du Chat (Cri du Chat syndrome, CdCS) est une anomalie chromosomique résultant d ’ une délétion de taille variable de l’extrémité du bras court du chromosome 5 (5p), incluant une région critique située en p15.2 [1]. Il représente une des délétions chromosomiques les plus fréquentes, son incidence dans la population générale est de 1/20 000 à 1/50 000 [1]. Les caractéristiques cliniques comprennent un cri monochromatique aigu, une microcéphalie, une dysmorphie cranio-faciale caractéristique évoluant avec l ’ âge et un retard mental et psychomoteur important [1]. La taille de la délétion est variable, Le traitement est fonction des différents symptômes. Un remaniement chromosomique parental est retrouvé dans 12% des cas et la majorité des délétions responsables de la maladie du cri-du-chat surviennent de novo [2]. Nous présentons une observation d ’ un syndrome du Cri du Chat, confirmé par caryotype métaphasique (46,XY, del(5)(p13) de novo). A travers cette observation nous mettrons à jour, les actualités scientifiques de ce rare syndrome, ainsi que la place des explorations cytogénétiques dans le diagnostic précis et le conseil génétique des syndromes dysmorphiques.

## Observation

**Figure 1:**
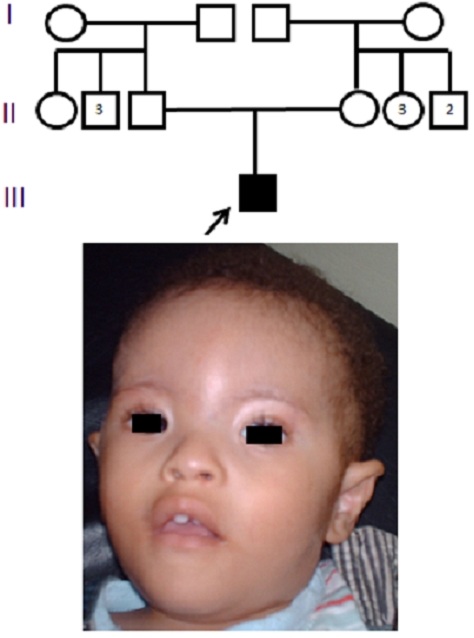
Arbre généalogique et aspect facial de notre patient présentant de Le syndrome du Cri du Chat

Nourrisson âgé de 11 mois de sexe masculin (Figure 1), adressé en consultation de génétique médicale pour dysmorphie et retard psychomoteur, issu d ’ un mariage non consanguin, d ’ une grossesse non suivie estimée à terme, de parents jeunes (âge du père 26 ans, âge de la mère 23 ans). Accouchement par voie basse avec un poids de naissance à 2 kg. Notre patient présente au cours de notre consultation : une hypotonie, un retard des acquisitions psychomotrices (pas de position assise et un retard de langage), Le poids et la taille est de -2 DS. La dysmorphie faciale est faite d ’ une microcéphalie, un visage lunaire, une trigonocéphalie, un hypertélorisme, une micrognathie, une racine du nez très large et plate, un épicanthuse et d ’ un strabisme. Les différentes explorations ont mis en évidence, une atrésie de l’oesophage type III (opère au cours de la première semaine de vie avec bonne évolution), une ectopie testiculaire bilatérale et un rein en fer à cheval avec une Urétéro-hydronéphrose gauche. Les parents sont phénotypiquement normaux.

Le caryotype métaphasique est réalisé à partir de cultures de lymphocytes à 37°C pendant 72h. Les cellules sont bloquées en métaphase par la colchicine. Après un choc hypotonique au KCI, les mitoses sont fixées par un mélange méthanol/acide acétique. Les préparations chromosomiques ainsi obtenues sont dénaturées par la chaleur (bandes RHG) et colorées au Giemsa. Onze mitoses sont prises en photo, puis classées en fonction de la taille des chromosomes, de leur indice centromérique et de la succession des bandes, de façon semi-automatique grâce à un logiciel spécialisé couplé à une caméra.

Le caryotype métaphasique en bandes R de notre patient a mis en évidence la délétion 5p- (Figure 1): 46,XY,del(5)(p13) sur les 11 mitoses analysées. L’analyse du caryotype des parents (indispensable en cas d ’ anomalies de structure) est sans anomalies, donnant ainsi le caractère de novo de la délétion 5p-.

## Discussion

Le syndrome du Cri du Chat (Cri du Chat syndrome, CdCS) ou la délétion du bras court du chromosome 5(5p-), lorsqu’elle inclut une région critique située en p15.2, est responsable d ’ un syndrome bien caractérisé. Les premières observations cliniques et cytogénétiques sont décrites pour la première fois par Lejeune et collaborateurs en 1963 [1]. Les caractéristiques cliniques les plus importants sont un cri monochromatique aigu, une microcéphalie, une arête nasale large, un épicanthus, une micrognathie ainsi qu’un retard mental et psychomoteur important. La taille de la délétion peut aller de 5 à 40 Mb, incluant une région critique située en p15.2 [3,4].

L’incidence dans la population générale est de 1/20 000 à 1/50 000, ce qui est en fait une des délétions chromosomiques les plus fréquentes. La prévalence chez les sujets retardés mentaux avec un quotient intellectuel inférieur à 50 serait de l’ordre de 1/350 [1]. Le poids de naissance est inférieur à la moyenne dans 90% des cas, malgré une durée pratiquement normale de grossesse. Plus de la moitié des nouveau-nés ont un périmètre crânien inférieur au 10ème percentile. Des troubles respiratoires et d ’ alimentation sont communs en période néonatale. Le poids, la taille et le périmètre crânien restent inférieurs à la moyenne [1]. Le signe le plus remarquable est celui qui a donné son nom à la maladie : un cri monotone, aigu et plaintif, rappelant le miaulement d ’ un chaton. Ce cri est présent au cours des premières semaines de vie, et se modifie par la suite [5]. L’aspect du visage est très évocateur. Il évolue avec l’âge. Chez le nourrisson, microcéphalie, hypertélorisme, micrognathie sont des signes cliniques évocateurs. La racine du nez est très large et plate. Il existe fréquemment un épicanthus, et un strabisme. Avec l’âge, le visage devient long et mince avec effacement des angles de la mâchoire. L’hypotonie est constante en période néonatale et dans la petite enfance, mais disparaît ensuite. Les acquisitions sont retardées, la position assise est acquise après 2 ans et la marche autonome rarement avant 4 ans. Le langage reste le plus souvent réduit à quelques mots, ou inexistant. Le retard mental est évident dès les premiers mois. Il est sévère à profond [3].

Il peut exister des malformations mineures, accessibles au traitement médical ou chirurgical, telles que strabisme, malocclusion dentaire, reflux gastro-oesophagien, pieds-bots, hernie inguinale, fentes labiale ou palatine et luxation de hanche. Les problèmes médicaux les plus fréquents au cours de l’enfance sont les infections des voies respiratoires supérieures, les otites moyennes, une constipation sévère et l’hyperactivité avec automutilation. Les scolioses sont relativement fréquentes au-dessus de 8 ans [3]. Les malformations viscérales graves sont rares, et s’observent le plus souvent en cas de translocation déséquilibrée. Il s’agit surtout de cardiopathies et d ’ anomalies du tube digestif. Les anomalies urogénitales sont rares, représentées par des cryptorchidies et hypogonadisme [6]. Des ectopies et agénésies rénales, ou des reins en fer à cheval ont été décrits. Le développement sexuel est normal pour les deux sexes et un seul cas de maternité a été rapporté dans la littérature mondiale [3]. De graves problèmes du comportement, tels que l’automutilation, agressivité et des mouvements stéréotypés, ont été rapportés dans plusieurs observations [7].

Le caryotype métaphasique (bandes R et/ou G) confirme le diagnostic. La taille de la délétion est variable allant de la totalité du 5p à une délétion limitée à la région 5p15.2-15.3. Elle est habituellement présente dans toutes les cellules, bien que quelques cas de mosaïque soient connus L’anneau du chromosome 5 et les translocations non équilibrées de novo ont également été rapportés. Dans quelques cas, un caryotype prométaphasique et/ou une étude par FISH (hybridation in situ fluorescente sur préparations chromosomiques (FISH : Fluorescence In Situ Hybridization)(hybridation in situ fluorescente sur préparations chromosomiques (FISH: Fluorescence In Situ Hybridization) avec une sonde spécifique de la région seront nécessaires pour identifier une très courte délétion, ou pour analyser plus précisément une délétion du bras court du chromosome 5 s’accompagnant d ’ un tableau clinique inhabituel [1]. Des techniques récentes, telles que CGH array (comparative genomic hybridization array) et PCR quantitative (Polymerase Chain Reaction quantitative), principalement utilisées dans le domaine de la recherche, permettent de caractériser des points de cassures et des microremaniements [3].

Le syndrome du Cri du Chat est une entité clinique bien définie, mais caractérisée par une grande variabilité phénotypique, expliquée par la variabilité de la taille de la délétion allant de la totalité du 5p à une délétion limitée à la région 5p15.2-15.3. [1]. La région critique pour la maladie du cri-du-chat (CDCCR, Cri Du Chat Critical Region), est une région d ’ environ 2Mb située en 5p15.2, la taille moyenne des délétions est de 5 à 40 Mb, et englobe largement cette bande. Le retard mental et la croissance sont en partie fonction de la taille de la délétion [1]. Des graphiques modélisant la croissance spécifique et le développement psychomoteur ont été établis. Deux gènes, Semaphorine F (SEMAF) et d-catenine (CTNND2), localisés dans les « régions critiques », sont potentiellement impliqués dans le développement cérébral et leur délétion pourrait être associée au déficit intellectuel chez les patients atteints du SCdC. La délétion du gène de la télomérase reverse transcriptase (hTERT) localisé en 5p15.33 pourrait contribuer aux manifestations phénotypiques observées chez les patients [3].

La majorité des délétions responsables de la maladie du cri-du-chat surviennent de novo, probablement au cours de la gamétogenèse. Il n’existe pas de facteur causal connu. L’âge parental moyen n’est pas augmenté. Dans 10 à 15% des cas, le chromosome anormal est transmis par l’un des parents, porteur d ’ une translocation équilibrée impliquant le 5 et un autre chromosome, ou, plus rarement, d ’ une inversion péricentrique du 5, ou d ’ une mosaïque parentale [2]. Le caryotype des parents est nécessaire. Le conseil est rassurant si le caryotype des parents est normal. En cas de translocation parentale, le diagnostic anténatal ou préimplantatoire (DPI) est possible [1]. Le traitement est fonction des différents symptômes. La prise en charge des troubles psychomoteurs, par une rééducation précoce (kinésithérapie, psychomotricité, orthophonie) a amélioré le pronostic de façon significative [3].

**Figure 2:**
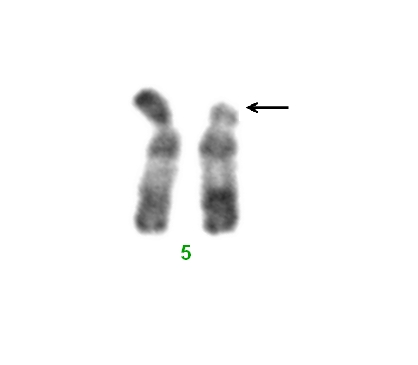
Le caryotype partiel métaphasique en bandes R de notre patient a mis en évidence la délétion 5p-:46,XY,del(5)(p13) (La flèche indique le niveau de la délétion)

L’espérance de vie est élevée et la morbidité est faible après les premières années de la vie. La mortalité survient le plus souvent au cours des premiers mois de vie, en rapport avec des malformations viscérales. Trois patients rapportés ont vécu plus de 50 ans [1]. Nos rapportons la première observation de notre unité de génétique médicale, d ’ un syndrome du Cri du Chat, confirmé par caryotype métaphasique : 46,XY,del(5)(p13) sur les 11 mitoses analysées (Figure 2). Notre patient âgé de 11 mois présente des signes rapportés dans ce syndrome et qui sont une hypotonie, un retard des acquisitions psychomotrices, une microcéphalie, un visage lunaire, un hypertélorisme, une micrognathie, un épicanthus, un strabisme, une atrésie de l’œsophage type III, une ectopie testiculaire bilatérale et un rein en fer à cheval rarement décrit. Notre patient est décédé 1 mois après le diagnostic, dans un tableau d ’ infection urinaire et d ’ altération de l’état général. Il s’agit d ’ un couple jeune, le conseil est rassurant puisque la délétion 5p est de novo (caryotype parental est normal). Néanmoins un suivi obstétrical et un diagnostic anténatal cytogénétique est souhaitable.

## Conclusion

La délétion du bras court du chromosome 5, lorsqu’elle inclut une région critique située en p15.2, est responsable d’un syndrome bien caractérisé, la maladie du Cri-du-Chat, incluant une dysmorphie cranio-faciale caractéristique qui évolue avec l’âge, le handicap mental dans la forme caractéristique est très sévère. Les malformations viscérales sont relativement rares et peu spécifiques. La taille de la délétion est variable. Nous rapportons l’observation type d ’ un syndrome de délétion 5p-, dont le diagnostic précis a permis de prodiguer au jeune couple un conseil génétique adéquat.
